# Editorial: Role of endogenous regulators of innate immunity in sepsis

**DOI:** 10.3389/fimmu.2025.1745170

**Published:** 2025-11-25

**Authors:** Haichao Wang, Xianzhong Meng, Daolin Tang, Yongming Yao

**Affiliations:** 1 The Feinstein Institutes for Medical Research, Northwell Health, Manhasset, NY, United States; 2 Department of Surgery, University of Colorado, Denver, CO, United States; 3 Department of Surgery, UT Southwestern Medical Center, Dallas, TX, United States; 4 Department of Medical Innovation Research, Chinese PLA General Hospital, Beijing, China

**Keywords:** hemichannel, extracellular vesicle, serum amyloid A, TREM2, IL-8, glycogenin-1, dopamine, scavenger receptor class B type I

Sepsis, a life-threatening syndrome arising from a dysregulated host response to infection, remains a leading cause of mortality and long-term morbidity worldwide. The profound immune dysfunction characterizing sepsis–oscillating between hyperinflammation and immunosuppression–underscores the critical need to unravel the intricate network of endogenous regulators that govern innate immunity during this complex condition. This Research Topic in *Frontiers in Immunology* presents a collection of seven research and five review articles that significantly advance our understanding of these endogenous mechanisms, offering new insights into sepsis pathophysiology, novel diagnostic biomarkers, and promising therapeutic avenues.

## Orchestrating cellular responses: channels, vesicles, and alarmins

Several contributions highlight the critical role of endogenous molecular entities in orchestrating cellular communication and shaping the innate immune response. Li et al. reviewed Connexin 43 and Pannexin 1 hemichannels in macrophages, revealing how bacterial lipopolysaccharide (LPS) and serum amyloid A (SAA) upregulate these channels, leading to ATP efflux. This efflux intensifies inflammasome activation, pyroptosis, and the release of pathogenic damage-associated molecular patterns (DAMPs) like high mobility group box 1 (HMGB1), thereby fueling the inflammatory cascade. Crucially, the authors demonstrate that mimetic peptides targeting these hemichannels can modulate their activity and impact sepsis-induced lethality, positioning them as significant therapeutic targets.

Expanding on intercellular communication, You et al. reviewed the pathogenic and therapeutic roles of extracellular vesicles (EVs) in sepsis. EVs, released by both host cells and bacteria, act as vital paracrine components, delivering bioactive materials that can either promote inflammatory responses or serve as tools for therapeutic cargo delivery. This dual role underscores their intricate involvement in both disease progression and tissue repair, suggesting engineered EVs as a novel strategy for diagnosis and targeted intervention. This concept is further elaborated by Jiao et al., who focused on tubular epithelial cell (TEC)-derived EVs carrying SAA1. This study uncovers a novel mechanism where these EVs exacerbate sepsis-associated acute kidney injury (SA-AKI) by promoting neutrophil extracellular trap (NET) formation via the TLR4/p38 MAPK pathway. Importantly, targeting EV secretion or SAA1 within TECs alleviates both renal and remote lung injury, indicating kidney-lung crosstalk and highlighting TEC-EVs/SAA1 as a potential prognostic index for SA-AKI.

The versatile protein SAA emerges as a recurrent endogenous regulator across the topic. Mohanty et al. delved into the specific roles of SAA proteins in sterile and infectious diseases, demonstrating their dual functionality. SAA amplifies cytokine and chemokine responses during sterile inflammation (where SAA-deficient mice showed better survival) but is essential for bacterial clearance in infectious conditions (where SAA-deficient mice were more susceptible). This intricate balance is mediated via NF-κB signaling, highlighting SAA as a key modulator whose context-dependent actions are critical. The explicit link between SAA and hemichannels, and SAA1 carried by TEC-EVs, illustrates the complex interplay and functional convergence of these endogenous mediators.

## Decoding immune cell dynamics and dysfunction

The heterogeneity of immune cell responses and their impact on patient outcomes is a central theme. Chen et al. identified a specific subset of CD10-CD121b^+^ neutrophils in septic shock patients that correlated with severity and predicted immunosuppression. In these immunosuppressive neutrophils, CD121b blockade led to increased proinflammatory cytokine production. This identifies CD121b as both a predictive indicator and a therapeutic target, particularly as its expression is amenable to down-regulation by all-trans retinoic acid (ATRA).

In the context of specific organ injury, Shen et al. reviewed the TREM2 signaling pathway in sepsis-induced acute lung injury (ALI). TREM2, expressed predominantly on myeloid cells, plays a pivotal role in modulating inflammation. Understanding its functions and mechanisms in both physiological and septic lung injury scenarios, along with evaluating TREM2-targeted therapies, is crucial for addressing this severe septic complication. Complementing this, Wang et al. focused on lymphocyte subsets as predictors for SA-AKI. This study meticulously identified significant alterations in peripheral blood lymphocyte subsets – including various T cell activation/exhaustion markers and myeloid-derived suppressor cells (MDSCs) – that predicted the incidence and severity of SA-AKI. The developed predictive model, incorporating these immunological parameters with clinical data, offers a robust tool for early warning and clinical decision-making.

The search for precise diagnostic tools is also addressed. Herminghaus et al. investigated the diagnostic utility of IL-18 plasma levels in distinguishing abdominal from non-abdominal sepsis. While several inflammatory cytokines were elevated in both sepsis types, IL-18 showed moderate predictive accuracy, particularly for identifying non-abdominal sepsis when its level was below a certain threshold. It suggests IL-18 as a useful additional biomarker, highlighting the importance of understanding specific inflammatory signatures based on infection source.

## Metabolic and neuro-immune axes in sepsis

Beyond direct immune cell interactions, systemic physiological changes significantly impact innate immunity. The comprehensive analysis of metabolism-related genes (MRGs) in sepsis by Zheng et al. revealed critical metabolic-immune heterogeneity. By stratifying patients based on MRGs, this multi-omics study identified a high metabolic risk group characterized by a neutrophil-dominant and lymphocyte-suppressed immune landscape. Glycogenin-1 (GYG1) emerged as a key hub gene, highly expressed in monocytes and neutrophils, and its knockdown significantly improved survival in a murine sepsis model. This positions GYG1 as a metabolic driver of innate immune hyperactivation and a promising therapeutic target.

The often-overlooked neuro-immune axis receives attention from the review paper by Wang et al., which highlights dopamine as an endogenous regulator of innate immunity in sepsis. Traditionally known for its role in the central nervous system, dopamine is shown to significantly influence the immune response, specifically regulating aconitate decarboxylase 1 (ACOD1). This mini-review underscores the critical, yet under-explored, interplay between the nervous and immune systems in sepsis.

Furthermore, Guo et al. explored the role of scavenger receptor class B type I (SR-BI) in sepsis, linking glucocorticoid (GC) biology to precision steroid therapy. This perspective review sheds light on how SR-BI mediates the adrenal stress response, which is crucial for controlling inflammation. By understanding SR-BI’s mechanisms, the authors advocate for a precision medicine approach to GC therapy, reserving it for septic patients with adrenal insufficiency rather than broad application.

## Harnessing host defense for therapeutic gains

Finally, the topic introduces potential therapeutic strategies derived from endogenous host defense mechanisms. Chen et al. presented pro-dermcidin and its derivatives as potential therapeutics for lethal experimental sepsis. While not directly bactericidal, pro-dermcidin and derivatives protected against sepsis by reducing inflammation, decreasing bacterial counts, and activating autophagy and phagosome maturation. This highlights a novel approach using host-derived peptides to modulate immune responses and improve outcomes.

## Interconnections, synthesis, and future directions

Collectively, the papers within this special topic paint a picture of sepsis as a highly dynamic and heterogeneous syndrome, driven by a complex interplay of endogenous regulators. A key recurring theme is the dual nature of many of these regulators – molecules like SAA, EVs, and hemichannels can be both beneficial and detrimental depending on the context, timing, and concentration. The metabolic-immune axis, exemplified by GYG1, and the neuro-immune axis, highlighted by dopamine, represent crucial systemic influences that modulate innate immune responses. The emphasis on specific immune cell subsets (CD121b^+^ neutrophils, lymphocyte populations in SA-AKI) and organ-specific complications (ALI, AKI) underscores the importance of a nuanced understanding of immune dysfunction beyond a generic “inflammatory response”.

The clear connections between studies are particularly exciting: SAA’s dual role is further contextualized by its ability to upregulate hemichannels and its packaging into TEC-derived EVs to drive organ injury. EVs, generally explored in the review paper by You et al., gain specific pathogenic function through SAA1 in the context of AKI (3). The metabolic underpinnings of immune cell behavior likely intersect with the activation states and phenotypes described for neutrophils and lymphocytes.

Looking ahead, these investigations pave the way for several critical future directions. First, continued integration of multi-omics data with single-cell analyses will be essential to map the precise regulatory networks governing sepsis heterogeneity and to develop personalized diagnostic as well as prognostic tools. Second, translating these findings into precision therapies requires careful consideration of the context-dependent roles of endogenous regulators. Targeting hemichannels, engineered EVs, or metabolic drivers like GYG1 offers exciting possibilities, but their therapeutic applications will likely benefit from patient stratification based on identified biomarkers (e.g., CD121b, IL-18, lymphocyte subsets, metabolic risk scores). Third, further exploration of less-understood axes, such as the neuro-immune system (dopamine), will undoubtedly uncover additional endogenous regulators with therapeutic potential. Finally, moving these promising preclinical findings into robust clinical trials will be the ultimate test of their impact on improving patient outcomes in sepsis. This Research Topic represents a significant step forward in our quest to tame the dysregulated host response in sepsis by understanding and strategically modulating its endogenous regulators.

